# Long-Term Postpartum Outcomes of Insulin Resistance and *β*-cell Function in Women with Previous Gestational Diabetes Mellitus

**DOI:** 10.1155/2020/7417356

**Published:** 2020-02-26

**Authors:** Zhirong Miao, Honghua Wu, Liu Ren, Nan Bu, Lili Jiang, Huixia Yang, Junqing Zhang, Xiaohui Guo

**Affiliations:** ^1^Department of Endocrinology and Metabolism, Peking University First Hospital, Beijing 100034, China; ^2^Department of Gynecology and Obstetrics, Peking University First Hospital, Beijing 100034, China

## Abstract

**Aims:**

The objective of the present study was to explore the long-term postpartum glucose metabolism in women with previous GDM, and study the mechanism of hyperglycemia from gestation to postpartum by investigating the postpartum insulin resistance and insulin secretion.

**Methods:**

A total of 321 females with previous GDM were followed up once during 1- to 6-years postpartum. Characteristics during pregnancy, perinatal period, and postpartum were compared between postpartum NGT and hyperglycemic women. HOMA-IR and HOMA-*β* were used to assess insulin resistance and insulin secretion levels with different glucose statuses.

**Results:**

The prevalence of postpartum hyperglycemia had a fluctuant increase from 25.9% at 1 year, to 53.7% at 5 year. 75 g OGTT 2 hPG during pregnancy was an independent predictor of postpartum hyperglycemia with an OR of 2.15 (95% CI 1.245, 3.722) (*P*=0.006). After ROC analysis, the best equilibrium between sensitivity (70.3%) and specificity (60.4%) for 2 hPG was 9.03 mmol/L. HOMA-IR was increased in postpartum normal glucose tolerance (NGT), prediabetes, and T2DM (1.64 vs. 2.14 vs. 4.27, *P*=0.006). After ROC analysis, the best equilibrium between sensitivity (70.3%) and specificity (60.4%) for 2 hPG was 9.03 mmol/L. HOMA-IR was increased in postpartum normal glucose tolerance (NGT), prediabetes, and T2DM (1.64 vs. 2.14 vs. 4.27, *β* were used to assess insulin resistance and insulin secretion levels with different glucose statuses. *P*=0.006). After ROC analysis, the best equilibrium between sensitivity (70.3%) and specificity (60.4%) for 2 hPG was 9.03 mmol/L. HOMA-IR was increased in postpartum normal glucose tolerance (NGT), prediabetes, and T2DM (1.64 vs. 2.14 vs. 4.27, *β* were used to assess insulin resistance and insulin secretion levels with different glucose statuses.

**Conclusions:**

75 g OGTT 2h PG during pregnancy higher than 9.03 mmol/L is regarded as an independent risk factor of postpartum hyperglycemia. Insulin resistance with insufficient insulin secretion compensation is still common phenomenon during long-term postpartum. Women with heavier insulin resistance in the postpartum period are more likely develop prediabetes, while decreased *β*-cell function contributes more to T2DM development.*β* were used to assess insulin resistance and insulin secretion levels with different glucose statuses.

## 1. Introduction

Gestational diabetes mellitus (GDM) is a common metabolic disorder during pregnancy, which is usually diagnosed in the second and third trimesters of pregnancy and is not overt diabetes [1]. According to the estimation by IDF in 2017, the global prevalence of hyperglycemia during pregnancy is 16.2%, and 86.4% of these is due to GDM [2]. GDM increases the risk of perinatal adverse outcomes [3], such as large for gestational age (LGA), cesarean delivery, neonatal hypoglycemia, shoulder dystocia, hyperbilirubinemia, preeclampsia, etc. The adverse effects of GDM in mothers as well as offspring do not end with pregnancy termination. Several studies [4–8] have proved that both mothers and offspring have increased risk of metabolic disorders in the long term. Compared to women with normoglycemia during pregnancy, women with previous GDM are more likely to have the risk of developing postpartum type 2 diabetes mellitus (T2DM) (RR 7.43, 95% CI 4.79–11.51) [4]. This indicated that GDM might share similar pathogenesis with T2DM, and both are related to insulin resistance and insufficient *β*-cell function [9].

During midpregnancy, the insulin resistance progressively increases, and similarly the *β*-cell function also increases to remain in the euglycemic status. GDM develops in women when there is excessive insulin resistance, inadequate *β*-cell compensation, decreased *β*-cell function, or any combination of these conditions [9]. Although the remission of glucose intolerance is a frequent phenomenon after delivery, insulin resistance may not disappear completely postpartum [10, 11], eventually leading to T2DM. So, it is meaningful to investigate the changes in insulin resistance and secretion postpartum. As far as we know, most of the existing literature related to the research focused for a short time with a limited number of patients in the postpartum period [10–12]. So, it is necessary to follow-up more patients for a long time in the postpartum period to obtain a better understanding on this question. The present study aimed to use homeostatic model assessment-insulin resistance (HOMA-IR) and homeostatic model assessment-*β* (HOMA-*β*) to investigate insulin resistance and secretion during 1∼6 postpartum years of GDM.

## 2. Materials and Methods

### 2.1. Subjects

Between November 2006 and October 2013, a total of 1,191 pregnant women were diagnosed with GDM and underwent delivery at the Peking University First Hospital, and 1,134 having medical records during hospitalization were enrolled. From August 2013 to November 2014, all these 1,134 women were followed up by telephone, and 321(28.3%) of them finally agreed to come to the hospital once, for undergoing 75 g oral glucose tolerance test (OGTT) and other examinations. According to the results of 75 g OGTT, women with prediabetes or T2DM postpartum were analyzed as hyperglycemic. To further explore the variations in different blood glucose levels, the hyperglycemic group was conversely divided into prediabetes and T2DM groups.

This study was approved by the ethics committee of the Peking University First Hospital. All subjects provided written informed consent forms.

## 3. Methods

Before April 2011, the criteria put forwarded by the National Diabetes Date Group (NDDG) were used for diagnosing GDM. This method consisted of two steps, a 50 g glucose challenge test (GCT) and a 75 g OGTT during 24–28 weeks of gestation. If the 1-hour 50 g GCT glucose level was 7.8 mmol/L or higher, then a diagnostic OGTT was indicated. GDM was diagnosed if two or more values were equal to or exceeded the upper limits of normal: fasting: 5.8 mmol/L, 1 h: 10.6 mmol/L, 2 h: 9.2 mmol/L, and 3 h: 8.1 mmol/L, while gestational impaired glucose tolerance (GIGT) was diagnosed if there was only 1 higher value. In both tests, patients were instructed to fast for 12 h. 50 g or 75 g of glucose dissolved in 200–300 mL of water was administered within a 3 to 5 minutes period. As the concept of GIGT has been abandoned, GIGT in this study was defined as GDM.

After April 2011, the criteria of International Association of Diabetes and Pregnancy Study Groups (IADPSG) were used. A 75 g OGTT at 24–28 weeks of gestation was used in all women who were not previously diagnosed with overt diabetes or GDM. GDM was established if one or more values were equal to or higher than the following values, fasting: 5.1 mmol/L, 1 h: 10.0 mmol/L, and 2 h: 8.5 mmol/L.

At the same time, for all the women included in our study, fasting plasma glucose (FPG) should be less than 7.0 mmol/L, and 2-hour postprandial glucose (2 hPG) should be less than 11.1 mmol/L.

Postpartum abnormal glucose was measured by employing 75 g OGTT at the time of follow-up. T2DM was confirmed if FPG was ≥7.0 mmol/L or 2 hPG was ≥11.1 mmol/L. However, women with 2hPG between 7.8 mmol/L and 11.1 mmol/L were classified as having impaired glucose tolerance (IGT), and women with FPG between 6.1 mmol/L and 7.0 mmol/L had impaired fasting glucose (IFG). IFG or IGT were considered as prediabetes. Prediabetes and T2DM were classified into hyperglycemia.

### 3.1. Observation Indices

The observation indices included data during pregnancy, perinatal period, and at the time of follow-up. Maternal age, family history of DM, weight in early pregnancy, weight at delivery, weight change during pregnancy, FPG, 1 h postprandial glucose (1 hPG), 2 hPG, and insulin use during pregnancy were collected. Neonatal birth weight and feeding patterns were recorded. During follow-up, FPG, 2 hPG, fasting insulin (FINS), and 2-hour postprandial insulin (PINS) were measured. Follow-up age, weight, postpartum weight change, waist, hip, and waist hip ratio (WHR) were recorded. HOMA-IR = FPG (mmol/L) ^*∗*^FINS (uIU/ml)/22.5. HOMA-*β* = 20^*∗*^FINS (uIU/ml)/[FPG (mmol/L) −3.5]/100.

Plasma glucose levels were determined by glucose oxidase method. Insulin levels were measured by chemiluminescence.

### 3.2. Statistical Analyses

SPSS 21.0 was used for statistical analyses. Values were expressed as mean ± standard deviation (SD) for normally distributed measurement data, median (25% percentile, 75% percentile) for abnormally distributed measurement data, and number (percentage) for categorical variables. Comparisons were performed by independent-samples *t*-test, one-way ANOVA, nonparametric test, or chi-square test as appropriate. Multiple logistic regression (forward: likelihood ratio) was used to analyze the predictors of postpartum hyperglycemia. Receiver operating characteristic (ROC) analysis was used to evaluate the predictive power of identified predictors. *P* values < 0.05 were considered as statistically significant in most of the analyses. Bonferroni method was used to adjust the *α* level for multiple comparisons.

## 4. Results

### 4.1. Characteristics of Included and Excluded Women

After followed up by telephone, 321 (28.3%) of the total population came to the hospital and underwent 75 g OGTT once at different postpartum years, while the rest 813 (71.7%) women refused and were excluded from our postpartum study. Characteristics during pregnancy and perinatal period were compared between the included group and excluded group (Table 1). Age at the time of pregnancy and the proportion of positive family history of DM were similar between groups (*P*=0.051, 0.283, ). Compared to the included group, the women in excluded group had lower weight (60.9 vs. 62.3 kg, *P*=0.013) and BMI (23.2 vs. 23.7 kg/m^2^, *P*=0.005) during early pregnancy period, while the weight and BMI at delivery were similar (*P*=0.340, 0.478). 75 g OGTT FPG, 1 hPG, and 2 hPG showed no significant differences between groups (*P*=0.139, 0.597, 0.340), as well as the proportion of women using insulin to control blood glucose levels during pregnancy (*P*=0.138). As to offspring, sex and born weight of the excluded group were also similar to the included group (*P*=0.749, 0.690).

### 4.2. Prevalence of Postpartum Hyperglycemia in Included Woman

**Figure 1:**
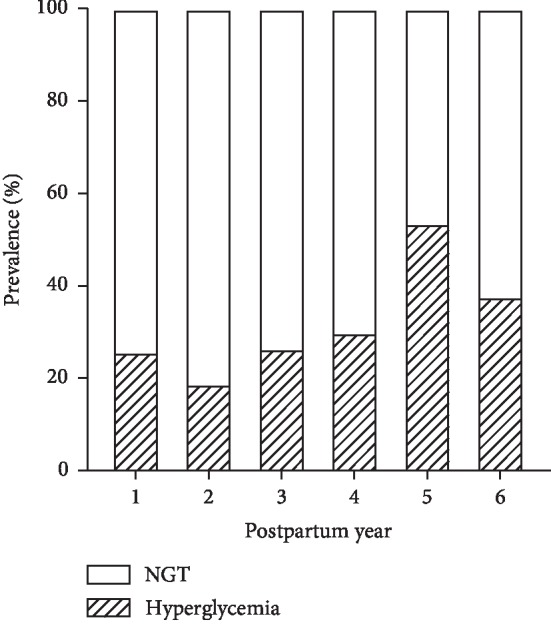
Prevalence of hyperglycemia in different years postpartum.

There were 54, 127, 34, 20, 41, and 45 women for 1-, 2-, 3-, 4-, 5- and 6-year postpartum study, respectively. 116 women diagnosed by NDDG criteria were followed up at 5 (4, 6)-year postpartum, and 205 women diagnosed by IADPSG criteria were followed up at 2 (1,2)-year postpartum. During the follow-up period, 92 (28.7%) women developed hyperglycemia, which included 16 (5.0%) T2DM women and 76 (23.7%) prediabetes women. From 1- to 6-year postpartum, there were, respectively, 14 (25.9%), 24 (18.9%), 9 (26.5%), 6 (30.0%), 22 (53.7%), 17 (37.8%) women diagnosed with hyperglycemia (Figure 1).

### 4.3. Characteristics of Postpartum NGT and Hyperglycemic Women

Compared to the NGT group, the hyperglycemic group included a slightly older women at the time of pregnancy, but the difference was not statistically significant (*P*=0.065, Table 2). The women in the hyperglycemic group more likely had a family history of DM than the women in the NGT group (31.5% *vs* 15.3%, *P*=0.001), and demonstrated to have a significantly higher weight (64.7 *vs* 61.5 kg, *P*=0.012) and BMI during early pregnancy period (24.6 *vs* 23.4 kg/m^2^, *P*=0.006). However, there were no significant differences in weight or BMI between the two groups at delivery (*P*=0.645, 0.578), and similarly in the neonatal birth weight (*P*=0.748). In the hyperglycemic group, more women used insulin to control blood glucose levels during pregnancy (37% vs 18.8%, *P*=0.001), and their 75 g OGTT 1 hPG and 2 hPG during pregnancy were significantly higher than those in the NGT group (*P* < 0.001), but FPG showed no significant differences (*P*=0.073). A greater proportion of women in the hyperglycemia group used formula to feed their offspring (20.4% *vs* 6.0%, *P*=0.034). At the time of follow-up, the maternal age, weight, BMI, waist, hip circumference, FPG, 2 hPG, FINS, and PINS were all higher in the hyperglycemic group (Table 2). But, WHR between the two groups showed no statistically significant differences (*P*=0.06). Weight change was smaller in the hyperglycemic group both during pregnancy (9.7 ± 3.8 *vs.* 12.0 ± 4.5 kg, *P* < 0.001) and in the postpartum period (−13.4 ± 5.4 *vs.* −10.3 ± 4.2 kg, *P* < 0.001). 8 (3.5%) women in the NGT group and 4 (4.3%) women in the hyperglycemic group had intervening pregnancies (*P*=0.748). Of all the intervening pregnancies, one woman completed delivery without GDM during the following pregnancy and developed NGT, another woman developed hyperglycemia at the time of follow-up with similar situation in the following pregnancy, and the rest of the women had abortion during early pregnancy.

### 4.4. Independent Risk Factors for Development of Hyperglycemia Postpartum

**Figure 2:**
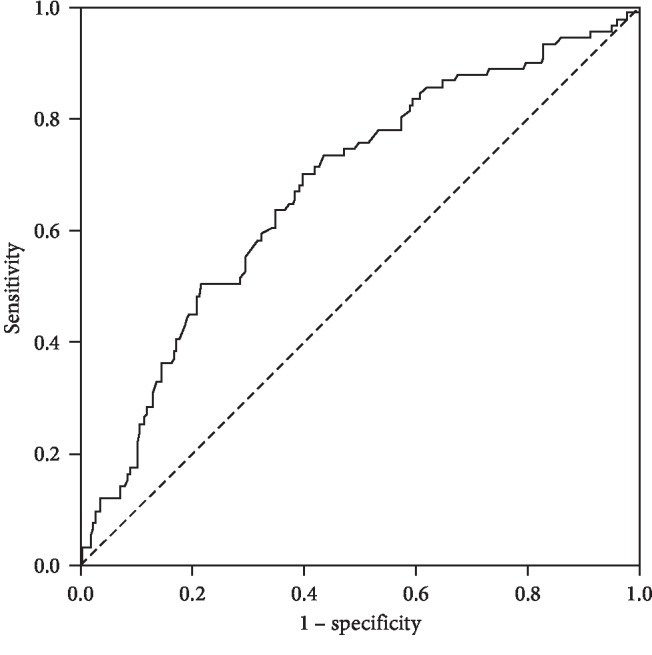
ROC curve for 75 g OGTT 2 hPG during pregnancy predicting postpartum hyperglycemia of GDM.

After excluding the collinearity indexes, maternal age, family history, weight during early pregnancy, insulin use, FPG, 1 hPG, 2 hPG, weight change during pregnancy, infant feeding, follow-up age, weight change during postpartum, maternal waist, and maternal hip circumference, women underwent logistic regression to identify independent risk factors of hyperglycemia postpartum. Finally, 75 g OGTT 2 hPG during pregnancy was demonstrated as an independent predictor of postpartum hyperglycemia with an OR of 2.15 (95% CI 1.245, 3.722) (*P*=0.006). ROC analysis was used to identify the optimal cut-off level of 2 hPG for predicting hyperglycemia postpartum (the area under the ROC curve = 0.674, 95% CI = 0.609–0.740, *P* < 0.001) (Figure 2). The best equilibrium between sensitivity (70.3%) and specificity (60.4%) for 2 hPG was 9.03 mmol/L. Positive predictive value (PPV) was 41.6%, and negative predictive value (NPV) was 83.5%.

### 4.5. HOMA-IR and HOMA-*β* in NGT and Hyperglycemic Women Postpartum

FPG and FINS during follow-up were used to calculate HOMA-IR and HOMA-*β*. HOMA-IR was 1.80 (1.20,2.62), and HOMA-*β* was 1.13 (0.79,1.60) in the overall population. HOMA-IR in the hyperglycemic group was significantly higher than that in the NGT group (2.36 (1.49,3.58) *vs* 1.64 (1.14,2.41), *P* < 0.001). HOMA-IR in the NGT, prediabetes, and T2DM groups was increased successively [1.64 (1.14, 2.41) *vs* 2.14 (1.49,3.22) *vs.* 4.27 (1.44,7.73), *P* < 0.001]. The differences between NGT and prediabetes, NGT, and T2DM showed significant differences (*P*=0.001, 0.001), while the difference was not statistically significant when comparing the pre-diabetes and T2DM groups after adjusting the *α* level (*P*=0.025). HOMA-*β* in the NGT group was higher than that in the hyperglycemic group (1.19 (0.84,1.62) *vs.* 1.04 (0.68,1.59)), but the difference was not significant (*P*=0.115). However, when the HOMA-*β* of NGT, prediabetes, and T2DM groups were calculated separately, the results were different [1.19 (0.84, 1.62) *vs* 1.11 (0.73, 1.66) *vs* 0.71 (0.42, 1.05), *P*=0.11]. Both HOMA-*β* of NGT and prediabetes groups were significantly higher than that of the T2DM group (*P*=0.003, 0.009), while the HOMA-*β* of the NGT group was not significantly higher than that of the prediabetes group (*P*=0.583).

## 5. Discussion

Due to increasing prevalence and adverse outcomes, especially during the high risk of metabolic disorders postpartum, GDM caused a huge economic burden to the society, and gradually became a serious public health problem. The risk of progression from GDM to T2DM varies with ethnicity, length of follow-up period, and cohort retention. Through systematic review, Kim C et al. [5] found that from 6-weeks to 28-years postpartum, the cumulative incidence of T2DM ranged from 2.6% to over 70%. After adjusting the confounding factors, the study further revealed that the progression to T2DM is steeply increased in the first 5 years postpartum, and then appeared to reach a plateau. In the present study, the prevalence of hyperglycemia with previous GDM had a fluctuant increase from 25.9% at 1-year postpartum, to 53.7% at 5-year postpartum. On the contrary, the decline of prevalence at 6-year postpartum might be related to the small sample size at this year. On the other hand, just like the progression to T2DM seemed to reach a plateau after first 5 years postpartum, the progression to hyperglycemia might have the same phenomenon, which might also contribute to decline of prevalence. Consistent with Kim C et al.'s study, our study also implied that 5-year postpartum is a critical period for the development of glucose metabolic disorder in women with previously diagnosed GDM. Intensive blood glucose monitoring during this period would be meaningful.

GDM is obviously considered as a risk factor for T2DM, but this phenomenon is not fully explained. Similar to T2DM, more and more studies [9, 13–15] showed that GDM also occurs due to insulin resistance and *β*-cell dysfunction. When various hormones and cytokines change with increasing gestation period, insulin resistance is progressively increased to the level of T2DM [9]. To remain euglycemic, *β*-cell function should be increased to compensate insulin resistance [9, 16]. GDM develops if maximal insulin secretion does not match with insulin resistance. In other words, gestational state is a challenge for *β*-cell function as well as systematic insulin resistance. It is known that both increased insulin resistance and inadequate insulin secretion contribute to hyperglycemia in nonpregnant women in varying degrees, and their roles in GDM are also different. According to a study [14], almost one-third women with GDM had insulin secretion defects without severe insulin resistance, one-half had predominant serious insulin resistance without insulin secretion defects, and less than 20% had both defects. One thing that needs to be mentioned is that no matter whether the insulin secretion is inadequate or insulin resistance is too severe, both were compared to normal pregnancy. So, even if one GDM women had insulin secretion defects, her insulin secretion level might still be higher than in her nonpregnant state.

As insulin resistance and insulin secretion defects contribute to both GDM and T2DM, the changes after delivery in the short term and long term are necessary to explain the transition from GDM to T2DM. In short-term studies [11, 17], insulin resistance remained higher in women with GDM previously, while their insulin secretion rates were similar to the previous nondiabetic pregnant women. Our study mainly focused on the long-term period. In women with previous GDM, the levels of HOMA-IR and HOMA-*β* were both still higher than 100% overall after delivery. This meant that they still had insulin resistance and insulin compensatory secretion, and some of their glucose levels were even recovered to normal. Within 6-year postpartum, the women who developed hyperglycemia had significantly higher levels of HOMA-IR than those with normal glucose levels, while the difference between prediabetes and T2DM was not significant. According to the HOMA-*β* levels, the normal glucose, prediabetes, and diabetes were decreased successively. In contrast to HOMA-IR, only the diabetes group demonstrated a significant decrease of HOMA-*β* than normal glucose and prediabetes. These results were in accordance with the study conducted by Ekelund M et al. [18]. This study further confirmed that the progression from GDM to T2DM may have similar pathogenesis to T2DM in other population groups. Along with increased insulin resistance, insulin secretion showed a compensatory increase. Prediabetes followed with further increase in insulin resistance. With excessive compensation of *β*-cell function, insulin secretion ultimately decreased, with consequent development of T2DM.

Since not all GDM women would develop hyperglycemia postpartum, the women who developed may have some characteristics during pregnancy and after delivery. In the previous studies [19, 20], prepregnancy body weight, gestational age at diagnosis, family history of diabetes, and larger area under the curve of glucose during antepartum OGTT were considered as independent risk factors of T2DM conversion. In our study, women who developed hyperglycemia postpartum more likely had a family history of DM, higher weight and BMI during early pregnancy, and higher 75 g OGTT 1 hPG and 2 hPG during pregnancy. They also require more insulin to control blood glucose levels, while the formula feeding was more common in this group. During the follow-up period, women with hyperglycemia had higher age, weight, BMI, and WHR, but their weight loss after delivery was relatively lower than that of NGT women. By multiple logistic regression analysis, 75 g OGTT 2 hPG during pregnancy was demonstrated to be an independent risk factor of postpartum hyperglycemia, with an OR of 2.153(95% CI,1.245–3.722). After ROC analysis, we found that the optimal cut-off level of 2 hPG was 9.03 mmol/L for predicting hyperglycemia postpartum. This meant that GDM women with higher OGTT 2 hPG during pregnancy, especially those higher than 9.03 mmol/L, should be closely monitored after delivery, and much efforts should be made to relieve them from insulin resistance through lifestyle changes, particularly within 5-year postpartum.

In our study, 28.3% of the total population accepted the postpartum 75 g OGTT. Women who refused postpartum tests had lower weight and BMI during early pregnancy period than the included women, which could cause some selection bias. However, other characteristics during pregnancy and perinatal period showed no significant differences, indicating that the included women had representativeness to a certain extent. Our study was retrospective in nature, experiencing the change of diagnostic criteria of GDM, women were followed up only once at different postpartum years, and lack of data between perinatal and follow-up time may lead to the difficulty of acquiring the exact incidence of hyperglycemia in different postpartum years. We could only get the prevalence, but the small sample size in some postpartum years might influence the accuracy. HOMA-IR and HOMA-*β* could reflect the levels of insulin resistance and insulin secretion during the course. As serum insulin might be influenced by many factors, HOMA2-IR and HOMA2-*β* [21, 22] based on C-peptide concentrations might show a better performance than insulin. So, a prospective and longer follow-up of women with previous GDM in larger population, and the test for serum C-peptide level is needed in order to better explore the transition of insulin resistance and insulin secretion postpartum, and to elaborate the mechanisms of transition from GDM to T2DM.

## 6. Conclusions

In conclusion, women with previous GDM are still at a greater risk of developing prediabetes and even T2DM in long-term postpartum. 75 g OGTT 2 hPG during pregnancy higher than 9.03 mmol/L is regarded as an independent risk factor of postpartum hyperglycemia. Entering postpartum stage, insulin resistance with insufficient insulin secretion compensation is still common phenomenon during long-term postpartum. Women with severe insulin resistance during postpartum are more likely to develop prediabetes, but decreased *β*-cell function contributes more to T2DM development.

## Figures and Tables

**Table 1:** Characteristics of the included and excluded groups.

Groups		Included	Excluded	Statistics	*P* value
	N	321	813		

During pregnancy	Maternal age, yr	32 (30,35)	31 (29,34)	3.814	0.051
	Family history of DM, n (%)	64 (19.9%)	140 (17.2%)	1.152	0.283
	NDDG criteria				
	GIGT	45 (38.8%)	174 (45.0%)	1.381	0.240
	GDM	71 (61.2%)	213 (55.0%)		
	Weight in early pregnancy, kg	62.3 ± 9.6	60.9 ± 10.3	−2.477	0.013
	BMI in early pregnancy, kg/m^2^	23.7 ± 3.3	23.2 ± 3.7	−2.812	0.005
	Insulin use, n (%)	77 (24.0%)	163 (20.0%)	2.198	0.138
	FPG, mmol/L	5.22 ± 0.61	5.22 ± 0.95	−1.479	0.139
	1 hPG, mmol/L	10.19 ± 1.74	10.21 ± 1.77	−0.529	0.597
	2 hPG, mmol/L	9.05 ± 1.73	8.88 ± 1.67	−0.954	0.340
	Weight at delivery, kg	73.6 ± 9.7	74.5 ± 10.5	−0.954	0.340

Perinatal period	BMI at delivery, kg/m^2^	28.1 ± 3.4	28.4 ± 3.7	−0.709	0.478
	Weight change during pregnancy, kg	11.4 ± 4.4	13.6 ± 5.4	−6.165	<0.001
	Sex of offspring, n (%)				
	Female	148 (46.8%)	363 (45.8%)	0.102	0.749
	Male	168 (53.2%)	430 (54.2%)		
	Born weight, g	3314.1 ± 478.6	3328.9 ± 533.6	−0.399	0.690

**Table 2:** Characteristics of NGT and hyperglycemic groups.

Groups		NGT	Hyperglycemia	Statistics	*P* value
	N	229	92		

During pregnancy	Maternal age, yr	32 (30,34)	32.5 (30,36)	−1.844	0.065
	Family history of DM, n (%)	35 (15.3%)	29 (31.5%)	10.841	0.001
	NDDG criteria				
	GIGT	29 (43.3%)	16 (32.7%)	1.347	0.246
	GDM	38 (56.7%)	33 (67.3%)		
	Weight in early pregnancy, kg	61.5 ± 9.0	64.7 ± 10.8	−2.535	0.012
	BMI in early pregnancy, kg/m^2^	23.4 ± 3.0	24.6 ± 3.9	−2.758	0.006
	Insulin use, n (%)	43 (18.8%)	34 (35.9%)	10.661	0.001
	FPG, mmol/L	5.18 ± 0.57	5.33 ± 0.70	−1.804	0.073
	1 hPG, mmol/L	9.84 ± 1.65	11.08 ± 1.65	−5.95	<0.001
	2 hPG, mmol/L	8.78 ± 1.61	9.72 ± 1.85	−4.500	<0.001
	Weight at delivery, kg	73.3 ± 9.3	74.5 ± 10.8	−0.997	0.319

Perinatal period	BMI at delivery, kg/m^2^	27.9 ± 3.2	28.4 ± 3.8	−1.171	0.241
	Weight change during pregnancy, kg	12.0 ± 4.5	9.7 ± 3.8	4.053	<0.001
	Sex of offspring, n (%)				
	Female	109 (48.4%)	39 (42.9%)	0.812	0.367
	Male	116 (51.6%)	52 (57.1%)		
	Born weight, g	3321.4 ± 476.9	3295.8 ± 484.9	0.430	0.667
	Infant feeding, n (%)				
	Lactation	40 (48.2%)	20 (37%)	6.770	0.034
	Formula feeding	5 (6.0%)	11 (20.4%)		
	Mixed feeding	38 (45.8%)	23 (42.6%)		

Follow-up time	Maternal age, yr	35 (32,38)	36.5(34,40)	−3.316	0.001
	Maternal weight, kg	60.0 ± 9.2	64.0 ± 10.6	−3.232	0.001
	Maternal BMI, kg/m^2^	22.8 ± 3.2	24.5 ± 3.9	−3.734	<0.001
	Weight change postpartum, kg	−13.4 ± 5.4	−10.3 ± 4.2	−5.196	<0.001
	Maternal waist, cm	79.71 ± 8.02	83.63 ± 10.50	−3.222	<0.001
	Maternal hip circumference, cm	95.01 ± 6.48	97.99 ± 6.92	−3.653	<0.001
	Maternal WHR	0.84 ± 0.05	0.85 ± 0.06	−1.886	0.06
	FPG, mmol/L	4.88 (4.59,5.13)	5.28 (4.85,5.82)	−5.415	<0.001
	2hPG, mmol/L	6.10 (5.37,6.85)	8.91 (8.25,10.33)	−13.170	<0.001
	FINS, uIU/ml	7.55 (7.42,11.16)	9.62 (6.17,13.41)	−2.933	0.003
	PINS, uIU/ml	54.83 (34.40,77.10)	87.88 (54.02,119.50)	−5.924	<0.001
	Intervening pregnancies	8 (3.5%)	4 (4.3%)		0.748

NGT, normal glucose tolerance; DM, diabetes mellitus; NDDG, National Diabetes Data Group; GIGT, gestational impaired glucose tolerance; GDM, gestational diabetes mellitus; BMI, body mass index; FPG, fasting plasma glucose; 1 hPG, 1 hour postprandial glucose; 2 hPG, 2 hour postprandial glucose; WHR, waist hip ratio; FINS, fasting insulin; PINS, 2 hour postprandial insulin.

## Data Availability

The data used to support the findings of this study are included within the article.

## References

[1] American Diabetes Association (2007). Standards of medical care in diabetes. *Diabetes Care*.

[2] Nam Han Cho J. K., Claude J., Mbanya K. O. (2017). *IDF Diabetes Atlas Eighth Edition*.

[3] Metzger B. E., Buchanan T. A., Coustan D. R. (2007). Summary and recommendations of the fifth international workshop-conference on gestational diabetes mellitus. *Diabetes Care*.

[4] Bellamy L., Casas J.-P., Hingorani A. D., Williams D. (2009). Type 2 diabetes mellitus after gestational diabetes: a systematic review and meta-analysis. *The Lancet*.

[5] Kim C., Newton K. M., Knopp R. H. (2002). Gestational diabetes and the incidence of type 2 diabetes: a systematic review. *Diabetes Care*.

[6] Garcia-Vargas L., Addison S. S., Nistala R., Kurukulasuriya D., Sowers J. R. (2012). Gestational diabetes and the offspring: implications in the development of the cardiorenal metabolic syndrome in offspring. *Cardiorenal Medicine*.

[7] Damm P., Houshmand-Oeregaard A., Kelstrup L. (2016). Gestational diabetes mellitus and long-term consequences for mother and offspring: a view from Denmark. *Diabetologia*.

[8] Zhu Y., Zhang C. (2016). Prevalence of gestational diabetes and risk of progression to type 2 diabetes: a global perspective. *Current Diabetes Reports*.

[9] Fasshauer M., Blüher M., Stumvoll M. (2014). Adipokines in gestational diabetes. *The Lancet Diabetes & Endocrinology*.

[10] Hiroko K., Daisuke T., Akihiro H. (2015). Sustained decrease of early-phase insulin secretion in Japanese women with gestational diabetes mellitus who developed impaired glucose tolerance and impaired fasting glucose postpartum. *Japanese Clinical Medicine*.

[11] Homko C., Sivan E., Chen X., Reece E. A., Boden G. (2001). Insulin secretion during and after pregnancy in patients with gestational diabetes Mellitus1. *The Journal of Clinical Endocrinology & Metabolism*.

[12] Wei J., Li X., Gao J. (2015). Insulin secretion and tolerance of women with different gestational glucose regulation one year postpartum. *International Journal of Clinical & Experimental Medicine*.

[13] Jang E.-H., Kwon H.-S. (2013). *β*-Cell dysfunction and insulin resistance in gestational glucose intolerance. *The Korean Journal of Internal Medicine*.

[14] Powe C. E., Allard C., Battista M.-C. (2016). Heterogeneous contribution of insulin sensitivity and secretion defects to gestational diabetes mellitus: table 1. *Diabetes Care*.

[15] Ryan E. A., O’Sullivan M. J., Skyler J. S. (1985). Insulin action during pregnancy: studies with the euglycemic clamp technique. studies with the euglycemic clamp technique. *Diabetes*.

[16] Butler A. E., Cao-Minh L., Galasso R. (2010). Adaptive changes in pancreatic beta cell fractional area and beta cell turnover in human pregnancy. *Diabetologia*.

[17] Saucedo R., Zarate A., Basurto L. (2011). Relationship between circulating adipokines and insulin resistance during pregnancy and postpartum in women with gestational diabetes. *Archives of Medical Research*.

[18] Ekelund M., Shaat N., Almgren P., Groop L., Berntorp K. (2010). Prediction of postpartum diabetes in women with gestational diabetes mellitus. *Diabetologia*.

[19] Jang H. C. (2011). Gestational diabetes in korea: incidence and risk factors of diabetes in women with previous gestational diabetes. *Diabetes & Metabolism Journal*.

[20] Kwak S. H., Choi S. H., Jung H. S. (2013). Clinical and genetic risk factors for type 2 diabetes at early or late post partum after gestational diabetes mellitus. *The Journal of Clinical Endocrinology & Metabolism*.

[21] Ahlqvist E., Storm P., Käräjämäki A. (2018). Novel subgroups of adult-onset diabetes and their association with outcomes: a data-driven cluster analysis of six variables. *The Lancet Diabetes & Endocrinology*.

[22] Zou X., Zhou X., Zhu Z., Ji L. (2019). Novel subgroups of patients with adult-onset diabetes in Chinese and US populations. *The Lancet Diabetes & Endocrinology*.

